# Exploratory Analysis of Cerebrospinal Fluid IL-6 and IL-17A Levels in Subcortical Small-Vessel Disease Compared to Alzheimer’s Disease: A Pilot Study

**DOI:** 10.3390/diagnostics15060669

**Published:** 2025-03-10

**Authors:** Georgios Liakakis, Aigli G. Vakrakou, Fotini Boufidou, Vasilios Constantinides, Georgios Velonakis, George P. Paraskevas, Leonidas Stefanis, Elisabeth Kapaki

**Affiliations:** 1 Neurochemistry and Biological Markers Unit, 1st Department of Neurology, School of Medicine, Eginition Hospital, National and Kapodistrian University of Athens, Vass. Sophias Ave. 74, 11528 Athens, Greece; liakakisneurologist@gmail.com (G.L.); fboufidou@med.uoa.gr (F.B.); vassilis.kon@hotmail.com (V.C.); 2 Laboratory of Neuroimmunology, First Department of Neurology, Aeginition Hospital, National and Kapodistrian, University of Athens, 21 Papadiamantopoulou, Ilisia, 11528 Athens, Greece; avakrakou@med.uoa.gr; 3 Research Unit of Radiology, 2nd Department of Radiology, Medical School, “Attikon” University General Hospital, National and Kapodistrian University of Athens, Rimini 1, Chaidari, 12462 Athens, Greece; 4 2nd Department of Neurology, School of Medicine, National and Kapodistrian University of Athens, “Attikon” University General Hospital, Rimini 1, 12462 Athens, Greece; geoprskvs44@gmail.com; 5 1st Department of Neurology, School of Medicine, Eginition Hospital, National and Kapodistrian University of Athens, 74 Vass. Sophias Ave., 11528 Athens, Greece; lstefanis@bioacademy.gr

**Keywords:** subcortical small-vessel disease, IL-6, IL-17A, cerebrospinal fluid, albumin quotient, ventricles, neuropsychological profiles, MRI, biomarkers

## Abstract

**Background/Objectives**: Low-grade inflammation in the form of microglial activation may be involved in neurodegenerative and vascular dementias. Subcortical small-vessel disease (SSVD) is the main form of vascular dementia, associated with brain barrier dysfunction and endothelial and monocyte activation. IL-6 and IL-17A are known proinflammatory cytokines that contribute to the disruption of blood–brain barrier integrity and microvascular dysfunction, features that are central to SSVD pathophysiological pathways. We herein compared cerebrospinal fluid (CSF) IL-6 and IL-17A concentrations in SSVD and AD patients as well as control subjects and examined the potential associations among IL-6 and IL-17A levels with cognitive and ΜRΙ changes. The albumin quotient (Qalb) was also calculated. **Methods**: CSF IL-6 and IL-17A (18 SSVD, 17 AD, and 12 healthy controls) were measured with solid-phase sandwich ELISAs, while albumin levels were measured by immunonephelometry. MMSE, FAB, and the CLOX tests were used for cognitive assessment and MRI was used for atrophy and white matter hyperintensities. **Results**: Significantly elevated CSF levels of Qalb and IL-6 were found in SSVD patients compared to both AD (*p* = 0.02) and controls (*p* = 0.002), respectively. Moreover, CSF IL-6 levels displayed a significant inverse correlation with CLOX2 scores (r = −0.641, *p* = 0.02), as well as a positive correlation with the total normalized CSF volume (r = 0.7, *p* = 0.01). CSF IL-17A levels were found to be reduced in SSVD patients, compared to controls and AD patients (*p* < 0.0001 and *p* = 0.002, respectively). The IL-6/IL-17A ratio with a cut-off value > 1.004 displayed a sensitivity of 83.33% (95%CI; 60.78% to 94.16%) and a specificity of 68.97% (95%CI; 50.77% to 82.72%) for the discrimination of SSVD from AD patients and controls. **Conclusions**: In the present pilot single-center study, we found increased CSF IL-6 and IL-6/IL-17A ratio levels in SSVD patients that correlated with reduced scores in the CLOX2 test and increased CSF volume. These preliminary findings deserve further evaluation in larger cohorts in order to elucidate their potential as surrogate biomarkers for the discrimination of SSVD from AD pathology.

## 1. Introduction

Subcortical small-vessel disease (SSVD) is a frequent cause of vascular dementia (VaD), which is homogeneous and often under-recognized [1]. The term SSVD encompasses several underlying vascular pathologies, namely arteriolosclerosis, fibroid necrosis, lipohyalinosis, edema, and damage of the blood–brain barrier (BBB). The pathological features of SSVD involve small-vessel degeneration, lacunar infarctions, multiple subcortical microinfarcts, and white matter lesions extending into deep white matter. These lesions can be visualized with brain computed tomography (CT) and more accurately with brain magnetic resonance imaging (MRI) [2]. SSVD frequently coexists or favors the development of AD [3,4]. To this end, biomarkers reflecting distinct pathologies are necessary; both on a clinical level, for the optimal treatment of individual patients and on a research level, for the optimal stratification of patients in clinical trials.

Clinically, SSVD manifests with overlapping features of Binswanger’s disease and the lacunar state, including motor impairment, cognitive decline (particularly in attention, executive function, and information processing), and urogenital symptoms such as urinary urgency and incontinence [2].

On a molecular level, endothelial dysfunction and increased BBB permeability lead to the extravasation of plasma proteins, including fibrinogen, which activates microglia and astrocytes, promoting neuroinflammation [5]. This process contributes to the production of reactive oxygen species (ROS), proinflammatory cytokines, and matrix metalloproteinases (MMPs), further exacerbating tissue damage [6,7]. Notably, circulating monocytes in patients with progressive SSVD exhibit an inflammatory phenotype, with increased cytokine production capacity, including elevated secretion of IL-6 and IL-1β by an intermediate monocyte subset. Although circulating cytokines are associated with VaD, their precise role in determining disease risk remains unclear [8].

IL-6 is a pleiotropic cytokine that plays a crucial role in the innate immune response, especially during infection and sterile inflammation. Serum IL-6 levels have been shown to correlate with brain atrophy, particularly in gray matter and hippocampal volume, as well as MRI white matter hyperintensities (WMHs) in elderly individuals [9]. On the other hand, studies in patients with mild cognitive impairment (MCI) and AD have shown no significant changes in serum or cerebrospinal fluid (CSF) IL-6 levels [10].

IL-17A, the signature cytokine of Th17 cells, is a key mediator of vascular inflammation and has been implicated in conditions such as vasculitis, arterial hypertension, and ischemic cardiovascular disease [11,12]. The role of IL-17A’s in neurodegenerative diseases, particularly AD, is still debated; however, it is postulated to promote inflammation in the vascular wall and contribute to coagulation and thrombosis [13]. On the contrary, studies in AD mouse models have shown that IL-17A does not exacerbate neuroinflammation but rather decreases soluble amyloid beta (Aβ) levels in the CSF and hippocampus [14]. Additionally, lower IL-17A levels have been observed in the plasma of AD patients compared to healthy controls, suggesting its complex role in the pathophysiology of neurodegenerative diseases [15].

Given their involvement in endothelial activation and microvascular injury, both IL-6 and IL-17A may be promising biomarkers for monitoring inflammation in VaD and SSVD particularly. However, to date, no studies have assessed the CSF levels of IL-6 and IL-17A in SSVD patients. To address this gap, we compared CSF IL-6 and IL-17A concentrations in patients with SSVD, patients with AD, and healthy controls. Furthermore, we investigated the associations among cytokine levels and cognitive function, as well as MRI markers, such as WMHs and ventricular enlargement in SSVD patients.

## 2. Materials and Methods

### 2.1. Patients and Methods

A total of 47 subjects were included in the present study (35 patients and 12 controls). This was a retrospective–cross-sectional study conducted on patients who were consecutively hospitalized without selection between 2013 and 2016 in the Neurodegenerative Disorders and Epilepsy Ward of the 1st Department of Neurology of the National and Kapodistrian University of Athens (NKUA), at Eginition Hospital.

All patients and/or relatives as well as healthy volunteers gave written informed consent for inclusion in this study, which was performed according to the 1964 Declaration of Helsinki and had the approval of the Scientific and Ethics Committee of our hospital.

Patients were clinically assessed independently by 3 clinicians (VC, GPP, and EK). Out of 35 patients, the following applies:

(a) Eighteen patients fulfilled the criteria for subcortical small-vessel disease, based on the International Society of Vascular and Cognitive Disorders (VASCOG) [16] and the Vascular Impairment of Cognition Classification Consensus Study (VICCCS) group [17]. Lacunar infarcts and WMHs primarily located subcortically were detected on MRI scans. Exclusion criteria for the study included the following: (i) patients with the presence of large artery disease defined as carotid artery stenosis (>50% assessed by carotid ultrasound), (ii) atrial fibrillation or the use of oral anticoagulants, (iii) previous cortical ischemic stroke or transient ischemic attack, (iv) intracranial hemorrhage (other than a microbleed), and (v) large artery vasculitis. To ensure pure SSVD pathology and exclude mixed pathology (SSVD and AD), the AD CSF biomarkers were measured. Normal results were among the inclusion criteria for the cohort of SSVD patients.
(b) Seventeen patients were diagnosed with AD according to the IWG-2 criteria, with biomarker support according to the AT(N) proposed framework [18,19]. The classification criteria implemented for a neurochemical AD diagnosis included an increase in CSF total (t-tau) and phosphorylated (p-tau) tau proteins, in addition to a decrease in the ratio of amyloid beta with 42 and 40 amino acids (Aβ42/40), based on the cut-off values of the Neurochemistry and Biomarker Unit of our department, as previously described [20].
(c) Additionally, 12 cognitively healthy controls who had undergone minor surgery (hernia repair or hip/knee joint surgery) were included for comparisons, and CSF samples were obtained during spinal anesthesia. The control group reported no cognitive symptoms and had no history of neurological, psychiatric, or other major illnesses. All control subjects had normal cognitive functions as assessed by a semi-structured interview and neuropsychological testing based on MMSE (Mini-Mental State Examination) and FAB (Frontal Assessment Battery). Moreover, in order to ensure non-underlying AD pathology, AD CSF biomarkers were also measured in the control group.
(d) In all patients, a battery of neuropsychological tests was performed, which included the following: (a) MMSE; (b) FAB; (c) the 5-word immediate and delayed recall (5WT); (d) the 15-point spontaneous and copy CLOX (clock drawing task) tests, CLOX 1 and 2, respectively [21,22,23,24].

### 2.2. CSF Handling and Analysis

CSF was obtained by lumbar puncture (LP) after overnight fasting at the L3–L5 interspace, according to recently proposed recommendations on standardized operating procedures for CSF biomarkers [25]. The CSF, collected in polypropylene tubes, was transferred to the lab within an hour and was centrifuged at 2000× *g* at 25 °C for 10 min. The ensuing supernatant was aliquoted in polypropylene tubes and stored at −80 °C, pending biochemical analyses.

Albumin levels in serum and CSF were measured by immunonephelometry. Blood was drawn at the same time point to calculate the serum levels of CRP, CHOL (cholesterol), HDL (high-density lipoprotein), LDL (low-density lipoprotein), and TRIG (triglycerides) to control for vascular risk factors and CSF/serum indices. The albumin quotient (Qalb) was calculated as CSF albumin (mg/L)/serum albumin (g/L), as previously described [26].

The IL-6 and IL-17A concentrations were assessed in a blind manner using an ELISA purchased from R&D Systems (Minneapolis, MN, USA). Briefly, uncoated 96-well plates were coated with primary antibodies overnight in an appropriate buffer, as indicated by the manufacturer. Wells were washed and blocked for 1 hr at room temperature (RT). Then, a 2-fold serial dilution of the top standards (recombinant IL-6 and IL-17A proteins) was placed in wells to make the standard curve for a total of 8 points (detection limit: 2 pg/mL). CSF from patients and controls was added undiluted in duplicate (100 λ of each sample) to the appropriate wells and incubated overnight at 4 °C. The next day, wells were thoroughly washed, treated with the biotin-conjugated secondary/detection antibody for 1 h at RT, and then coated with avidin–horseradish peroxidase for 30 min. After adding the substrate solution for 15 to 20 min, a stop solution was added to terminate the reaction (1 M H_3_PO_4_), and the plates were immediately red at 450 nm.

CSF biomarkers (t-Tau, p-Tau, Aβ42, and Aβ40) were measured in duplicate with ELISA by commercially available kits from EUROIMMUN and applied on the automated analyzer EUROIMMUN Analyzer I (EUROIMMUN, Medizinische Labordiagnostika AG, Lübeck, Germany), as previously described [20].

### 2.3. MRI Analysis

Neuroimaging examinations with an MRI of the brain were performed in all patients, according to previous protocols used [27]. We acquired 3D fluid-attenuated inversion recovery images and 3D T1-weighted images in the 2nd Neuroradiology Department of the NKUA. Volumetric data were extracted using the VolBrain (http://volbrain.upv.es, accessed on 24 October 2024) platform. VolBrain software contains advanced pipelines and automatically provides the volumetric information of the brain MRI images at different scales [28]. The brain was segmented into whole white matter, cortex, deep gray matter structures, and ventricles.

### 2.4. Statistical Analysis

Data were first tested for normal distribution. The Kruskal–Wallis test, a non-parametric equivalent of the One-Way ANOVA, was used to determine whether or not there was a statistically significant difference among the medians of the three independent groups followed by Dunn’s test for the correction of pairwise comparisons. A two-tailed *p*-value < 0.05 was considered statistically significant. To compute an approximate effect size, we used η^2^ (eta squared), a measure of the proportion of the variance explained by the independent variable. A group analysis of patients involved various clinical, biochemical, and radiological parameters as reported in the patients’ characteristics. Subsequently, to test the sensitivity and specificity of the tested biomarkers, receiver operating characteristic (ROC) curves were applied. Correlations were assessed by Spearman’s correlation coefficient. A statistical analysis was performed using GraphPad Prism-9.

## 3. Results

### 3.1. Characteristics of Individuals Included in This Study

The characteristics of the study subjects are shown in Table 1. The age and gender were similar among subgroups. No statistical difference was found between them.

**Figure 1:**
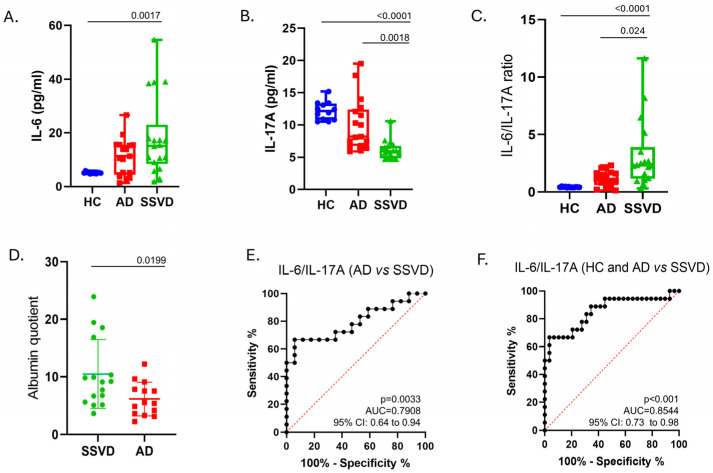
IL-6, IL-17A, and the ratio of IL-6/IL-17A in the CSF of SSVD and AD patients and controls. (**A**) CSF levels of IL-6 (Elisa, pg/mL), (**B**) CSF levels of IL-6 (Elisa, pg/mL), (**C**) CSF levels of IL-6/IL-17A ratio, (**D**) CSF (in mg/dL)/serum (in g/dL) albumin quotient, (**E**) ROC curve for differentiating AD vs. SSVD with IL-6/IL-17A ratio, (**F**) ROC curve for differentiating controls and AD vs. SSVD with IL-6/IL-17A ratio. IL-6: interleukin 6, IL-17A: interleukin 17A, SSVD: subcortical small-vessel dementia, AD: Alzheimer’s disease, HC: healthy controls, AUC: area under the curve, vs.: versus.

SSVD and AD patients recruited in this study had similar ages at disease onset and disease duration. The mean scores of the cognitive tests MMSE, FAB, and 5 words were significantly lower in patients with AD compared to SSVD (Table 1). The CSF albumin quotient was found to be increased in SSVD patients (Qalb; SSVD 10.47, AD 6.13, *p*-value 0.0199) (Table 2 and Figure 1D). No changes were found among groups concerning the serum levels of CRP, CHOL, HDL, LDL, and TRIG (Table 2). As expected, and described in the methods, SSVD presented a significantly higher Fazekas score compared to AD patients (Table 1).

### 3.2. Perturbations of IL-16 and IL-17A Protein Levels in CSF of SSVD, Compared to AD Patients and Controls

CSF IL-6 levels were increased in patients with SSVD compared to controls (*p* = 0.0017), whereas no significant differences were found in AD (Figure 1A). IL-17A levels were significantly lower in SSVD patients than in AD patients and the control group (*p* = 0.0018, *p* < 0.0001, respectively) (Figure 1B).

We then calculated the IL-6-to-IL-17A ratio and found that this is highly increased in SSVD, but not in AD patients, compared to controls (Figure 1C). This ratio could better discriminate SSVD from non-SSVD subjects with a cut-off value of >1.004, resulting in a sensitivity of 83.33% (95%CI; 60.78% to 94.16%) and a specificity of 68.97% (95%CI; 50.77% to 82.72%) (Figure 1E,F).

**Figure 2:**
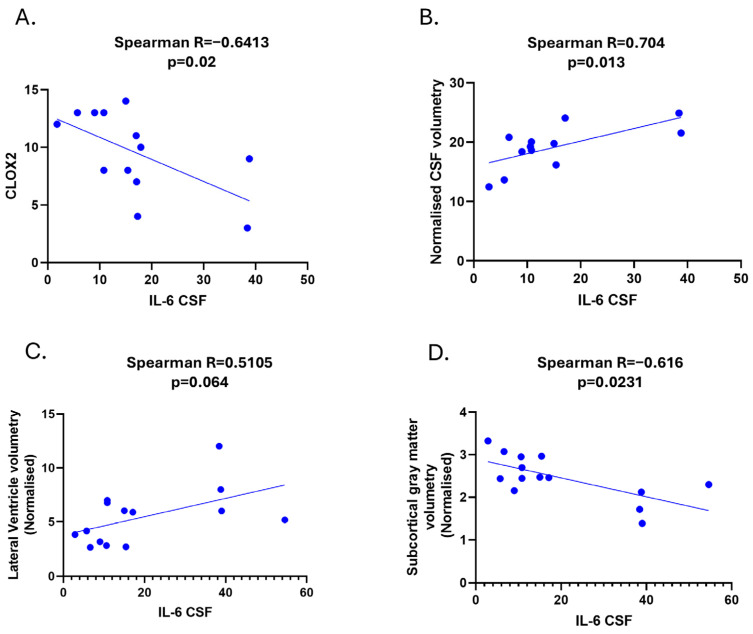
Clinical and radiological correlations of CSF IL-6 in SSVD. (**A**) Correlation among CSF IL-6 levels and CLOX2 cognitive test. (**B**) Correlation among CSF IL-6 levels and normalized CSF volumetry values form MRI volumetric data analysis. (**C**) Correlation among CSF IL-6 levels and normalized lateral ventricle volumetry values from MRI volumetric data analysis. (**D**) Correlation among CSF IL-6 levels and normalized subcortical gray matter volume from MRI volumetric data analysis (Volbrain software). SSVD: Subcortical small-vessel vascular dementia, CLOX: clock drawing task.

Regarding biochemical–neuropsychological correlations, a significant inverse correlation was found between CSF IL-6 levels and the CLOX2 score in SSVD (Spearman R; −0.6, *p* = 0.02) (Figure 2A). No other significant correlation was found with regard to neuropsychological data.

### 3.3. Cerebrospinal Fluid Levels of IL-6 Correlate with Lateral Ventricle Enlargements in MRI in the SSVD Group

We performed quantifications of WMHs (both in number and volume) as the Fazekas score is a semi-quantitative method for assessing WMHs. We also measured total gray matter volume, cortical and subcortical gray matter volume, as well as cortical thickness and ventricle enlargement (LesionBrain, Volbrain software).

We found that CSF IL-6 levels correlated with the normalized volume of CSF and especially with the volume of lateral ventricles, but not with the volumes of the third and fourth ventricle (Spearman R = 0.704, *p* = 0.013 and Spearman R = 0.51, *p* = 0.064, respectively) (Figure 2B,C).

**Figure 3:**
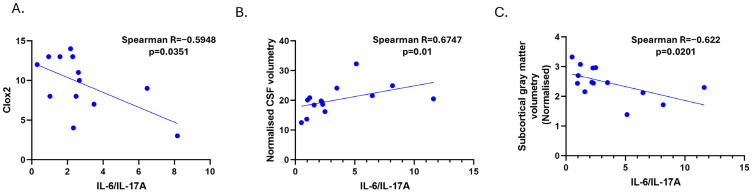
Correlations among CSF IL-6/IL-17A levels in SSVD patients with CLOX2 (**A**) and MRI parameters; normalized CSF volumetry (**B**) and subcortical grey matter volumetry (**C**). CLOX: clock drawing task, CSF: cerebrospinal fluid, and GM: gray matter.

We did not find any correlation between IL-6 in SSVD and WMHs (Fazekas score or the quantification of the volume of lesions). However, a significant negative correlation between IL-6 and the normalized subcortical gray matter volume was noticed (Spearman r = -0.616, *p* = 0.0231, Figure 2D). Significant correlations among IL-6/IL-17A ratio levels and CLOX2 and MRI parameters were also observed (Figure 3A–C).

On the other hand, AD patients were stratified into two groups based on Fazekas score (0–1 vs. 2 Fazekas score) and those with higher scores had increased CSF IL-6 levels (Supplementary Figure S1).

### 3.4. Discussion

Our study aimed to evaluate inflammatory cytokines CSF IL-6 and IL-17A in patients with vascular cognitive impairment of the subcortical small-vessel disease subtype in order to discriminate vascular from neurodegenerative causes of dementia, particularly AD. The latter can nowadays be diagnosed with biomarkers of molecular specificity, namely β-amyloid and p-tau protein, even in the asymptomatic phase of the disease [19]. In VaD, these markers are normal or slightly decreased regarding CSF Aβ42 levels, but this reduction is not evident when the Aβ42/40 ratio is calculated, as it is considered to correct for individual differences in amyloid production [17,20]. Abnormal values of the above biomarkers indicate the presence of AD co-pathology, which is very common in older ages. However, in addition to biomarkers for exclusion, there is an unmet need for the biomarkers of inclusion in VaD.

SSVD is the most homogeneous among VaD subtypes, and thus, an appropriate ground for biomarker research. Inflammation is constantly recognized to play an important role in dementia and in VaD particularly. Thus, molecules such as IL-6 and IL-17A in CSF, which are in close proximity with the brain, reflect the pathological changes taking place within. Accordingly, Dukic L et al. (2016), by studying AD, VaD, MCI, and cognitively healthy individuals found that serum IL-6 was significantly higher in VaD patients compared to AD, MCI, and cognitively healthy participants [29].

Wada-Isoe et al. (2004) reported significantly elevated CSF IL-6 levels in vascular dementia (VD) patients compared to those with AD or cerebrovascular disease without dementia [30]. Similarly, our study found increased CSF IL-6 levels in SSVD patients, further supporting the role of neuroinflammation in vascular cognitive impairment. A recent meta-analysis, including three studies, compared IL-6 levels in the CSF among 82 patients with VaD, 99 with AD, and 81 healthy subjects. IL-6 was significantly higher in VaD patients compared to healthy subjects but not compared to AD patients [31]. The authors commented that due to the high heterogeneity and poor overall quality of the studies, the reliability of these findings is limited. Our results align with previous studies, showing increased IL-6 in VaD compared to AD and controls, adding further experimental evidence for SSVD pathology, a distinct, homogeneous, and less-studied subtype of VaD.

IL-6 contributes to the shaping of microvascular walls, activating endothelial cells and leading to luminal narrowing and wall thickening. The endothelial dysfunction results in reactive gliosis, low-grade inflammation, and the loss of axonal integrity, which are some of the key pathophysiological mechanisms underlying SSVD. Another key mechanism, blood–brain barrier dysfunction, as indicated by an increased Qalb, was found in our study, in line with previous reports on SSVD patients [32,33]. However, this quotient did not correlate either with IL-6 or IL-17A protein levels.

We further showed that the CSF levels of IL-6 in patients with SSVD significantly and negatively correlated with the CLOX2 cognitive test. While this inverse correlation was statistically significant (Spearman R = −0.6, *p* = 0.02), the moderate strength of this association indicates that additional inflammatory or neurodegenerative mechanisms may also play a role in executive dysfunction and cognitive decline in SSVD, requiring further investigation. From a clinical perspective, patients with vascular dementia frequently present with early poor executive functioning [34]. The CLOX test in general constitutes a simple and effective tool to detect graphomotor/executive control dysfunction as well as perceptual/visuospatial deficits [24]. Clock drawing performance and disturbances in executive control function in some studies have been found more in the copy (CLOX2) than in the command condition (CLOX1) [35,36]. In the study of Margraf et al., 2009 controls performed significantly better than the subcortical ischemic vascular disease group (*p* = 0.001) in the CLOX2 test [37].

Cerebrovascular disease and vascular risk factors, particularly diabetes and hypertension, frequently result in SSVD, while they are also recognized as risk factors for AD [38]. The most common pathology in SSVD is diffuse damage to the white matter (ischemic leukoencephalopathy) [39]. In neurologically asymptomatic individuals, an increased serum IL-6 has been found in those with lacunes and silent brain infracts. In line with previous preclinical and clinical studies demonstrating a link between silent brain infracts and serum IL-6, we found that CSF IL-6 is positively correlated with lateral ventricle enlargement. Previous studies have shown that silent lacunar infracts were associated with atrophy in multiple subcortical structures, ventricular enlargement, and widespread cortical thinning [40]. We did not find an association between WMHs and CSF IL-6 levels possibly due to the already high score of SSVD patients in the Fazekas scale; however, we did find an association in the AD group (Supplementary Figure S1). The lack of correlation between CSF IL-6 and WMHs in SSVD was unexpected, given IL-6’s role in vascular pathology. One possible explanation is that SSVD patients in our study already had advanced white matter disease, as reflected by their high Fazekas scores, potentially creating a ceiling effect that limits the ability to detect further associations. Notably, IL-6 did correlate with ventricular enlargement and reduced subcortical gray matter volume, suggesting a stronger link to neurodegeneration in SSVD. In contrast, the correlation in AD may reflect distinct inflammatory mechanisms. Further longitudinal studies are needed to clarify these relationships.

Patients with SSVD displayed significantly lower CSF concentrations of IL-17A than both the control subjects and patients with AD. AD patients also had decreased CSF IL-17A levels compared to the control subjects. Previous studies have shown that CSF IL-17A levels in AD are not significantly altered compared to controls [41]. However, some studies suggest a peripheral increase in IL-17A in AD, particularly in serum, indicating potential compartmental differences in its regulation between the CNS and systemic circulation [42].

A possible explanation for this discrepancy between high IL-6 and low IL-17 levels in SSVD patients may be the different mechanisms required for Th17 cell differentiation as IL-6-dependent and IL-6-independent pathways play key roles. Moreover, IL-6 cannot induce Th17 cells on its own, as it must act synergistically with TGF β [43]. IL-17A has been extensively studied in autoantibody-mediated disease and classical chronic inflammatory diseases, such as neuromyelitis optica (NMO), rheumatoid arthritis, ankylosing spondylitis, and psoriasis [44,45]. The etiology of decreased IL-17A levels in CSF is still elusive. One explanation would be the lack of an inflammatory milieu with IL-21, TGFβ, or IL-23, which is known to induce the production of Th17 cytokines and promote autoimmunity in the presence of a cognate antigen, driving their maturation and maintenance [44,46]. In contrast to IL-6, which is a more classical indicator of innate immunity activation, IL-17A is more implicated in adaptive immune responses and in those specifically fueled by autoantibodies like AQ4. Interestingly, the inability to upregulate IL-17A could have a negative impact on decreasing pathological aspects related to plaque formation, especially in AD patients. This may explain the observed reduction in IL-17A in both SSVD and AD, as the lack of an adaptive immune response could impair potential neuroprotective functions in these conditions. The overexpression of IL-17A has been shown to decrease soluble Aβ levels without exacerbating neuroinflammation in a mouse model of Aβ accumulation [14]. Overall, our findings align with previous reports showing limited IL-17A involvement in neurodegenerative conditions, contrasting with its well-established role in autoimmune diseases where sustained Th17 activation is a hallmark [41].

Additionally, we found that the CSF IL-6/IL-17A ratio showed some potential in differentiating between controls and SSVD patients, with a less clear distinction between AD and SSVD. With a cut-off value of >1.004, the CSF IL-6/IL-17A ratio could discriminate SSVD from controls and AD patients, with high (over 80%) sensitivity (83.33%), but modest specificity (68.97%), according to the Working Group on Molecular and Biochemical Markers of AD, Reagan Research Institute of Alzheimer’s Association and National Institute on Aging (1998) [47]. Despite its modest specificity, the IL-6/IL-17A ratio was found to have a significantly inverse correlation with the CLOX2 score and a positive correlation with the normalized CSF volume in SSVD patients, (Figure 3). The underlying pathomechanisms of this correlation remain unclear; however, they may stem from the overlap of these cytokines with broader inflammatory processes not exclusive to SSVD Comorbidities could also influence cytokine levels, or they may indicate a distinct inflammatory milieu that influences disease severity [48,49]. However, the modest specificity raises doubts about the utility of this biomarker in clinical practice and, in any case, this finding warrants further validation in larger cohorts, longitudinal studies, and experimental models.

In the context of SSVD and AD, microglial activation plays a key role in the initiation and progression of both diseases [50,51]. Moreover, recent data have directly associated pericyte degeneration with significant white matter lesions, characterized by hypoxia and a loss of myelination, resulting in the disruption of anatomical and functional connections within the brain [52]. It is increasingly evident that SSVD as well as AD are complex disorders of neurovascular and neuroinflammatory underlying pathologies that require further investigation in both the onset of the disease and possible treatment approaches. For AD, a 2-hit vascular hypothesis model has been proposed by Zlokovic. The initial ’hit’ causes vascular impairment due to multiple risk factors, resulting in the disruption of vascular integrity, which contributes to cognitive decline and vascular dementia. The second ‘hit’ accounts for elevated Aβ levels (defective clearance), which worsens neuronal function and contributes additional damage to the pathogenesis of AD [53]. Overall, it is assumed that neuroinflammation in the form of glial cell activation due to stress factors (hypoxia, protein aggregation), including IL-6 signaling, propagates a vicious cycle and contributes to the final neuropathological changes.

Our study has certain limitations, which are as follows: (i) The size is small, being a single-center study; however, this study refers to the more homogeneous subtype of VaD, thus avoiding other problems resulting from heterogeneity in other VaD cohorts. (ii) Regarding AD, the lack of pathological confirmation is compensated by the use of CSF AD biomarkers, known to reflect in vivo AD pathological changes occurring in the brain with high accuracy [18]. Moreover, measuring CSF AD biomarkers in both SSVD and the control group, we avoid the contamination of these groups by AD, resulting in true controls (without yet asymptomatic AD) and pure SSVD samples (a disease condition that is relatively rare). (iii) The lack of matched serum samples for assessing the potential contribution of peripheral cytokine synthesis in CSF samples and the restricted number of cytokines measured, and (iv) the lack of the external validation of measurements.

## 4. Conclusions

In conclusion, the results of the present pilot study have shown an increase in CSF IL-6 levels and the IL-6/IL-17A ratio in SSVD patients compared to AD patients and controls, which deserve further evaluation in order to elucidate their potential role as surrogate biomarkers for the discrimination of SSVD from AD pathology. Furthermore, the correlation found between IL-6 and the CLOX2 test in patients with SSVD, but not in patients with AD, may reflect differences in the underlying inflammatory mechanisms of these two pathologically different types of dementia. Given the preliminary nature of our findings, future multicenter studies with larger cohorts and validation are required before the above-mentioned biomarkers might have clinical implications for the discrimination of SSVD from AD patients. Investigations for possible correlations with other cytokines, such as IL-1β and TNF-α, as additional links to elucidate the molecular pathology of these diseases could be of value.

## Figures and Tables

**Table 1:** Demographic, cognitive, and MRI data of patients and controls.

Subgroups	AD (*n* = 17)	SSVD (*n* = 18)	Controls (*n* = 12)	*p*-Value
Demographic				
Gender (M/F)	6/18 (33% M)	9/17 (53% M)	4/12 (33% M)	N/S
Age, y, (mean, SD)	69 (11.91)	72.56 (10.03)	74.70 (6.33)	N/S
Cognitive measurements				
MMSE	14.00 (4.56) ***	21.13 (5.94) **	28.2 (0.92)	*** *p* < 0.0001, ** *p* = 0.0067, * *p* = 0.049
FAB	6.88 (3.41) ***	10.60 (3.56) **	15.14 (1.58)	*** *p* < 0.0001, ** *p* = 0.0046 * N/S
5w in 1	1.438 (1.44)	3.25 (1.77)	N/A	0.004
5w in 2	0.75 (1.24)	1.13 (1.09)	N/A	NS
5w del 1	0.313 (0.70)	1.88 (1.78)	N/A	* *p* = 0.003
5w del 2	0.75 (1.13)	1.31 (1.30)	N/A	N/S
CLOX1	5.53 (3.98)	7.15 (4.47)	N/A	N/S
CLOX2	8.33 (3.48)	9.62 (3.53)	N/A	N/S
MRI analysis				
Scheltens scale	1.59 (0.79)	1.667 (1.39)	N/A	N/S
Fazekas scale	1.35 (0.61)	2.60 (0.63)	N/A	*p* < 0.0001

Abbreviations: SSVD: Subcortical small-vessel vascular dementia, AD: Alzheimer’s disease, y: years, MMSE: Mini-Mental State Examination, FAB: Frontal Assessment Battery, 5w: 5 words, del: delayed recall, CLOX: clock drawing task, N/S: Not significant, N/A: Not applicable. Comparison among two groups was performed with the Mann–Whitney U test, and comparison among three groups was performed with the Kruskal–Wallis test and Dunn’s correction for multiple comparisons test: *** AD compared to controls, ** SSVD compared to controls, and * AD versus SSVD. Comparisons of dichotomous variables were assessed by Chi-square. Data are represented as mean and SD (standard deviation).

**Table 2:** Serum and CSF analysis in AD and SSVD patients and the control group.

Subgroups	AD (*n* = 17)	SSVD (*n* = 18)	Controls (*n* = 12)	*p*-Values	Effect Size	Confidence Intervals
Serum analyses						
CRP (mg/dl)	0.098 (0.13)	0.28 (0.34)	N/A	N/S	N/A	N/A
CHOL (mg/dl)	200.67 (40)	203.7 (61.75)	N/A	N/S	N/A	N/A
HDL (mg/dL)	59.42 (15.73)	51.33 (17.57)	N/A	N/S	N/A	N/A
LDL (mg/dL)	123.83 (34.21)	127.8 (55.63)	N/A	N/S	N/A	N/A
TRIG (mg/dL)	108.17 (67.68)	127.29 (62.94)	N/A	N/S	N/A	N/A
CSF analyses						
Albumin Quotient (Qalb)	6.13 (2.91)	10.47 (5.99)	N/A	0.0199	N/A	−6.610 to −0.2800
IL-6 (pg/mL)	11.14 (6.87)	18.26 (14.67)	5.143 (0.31)	0.0026 * (controls vs. SSVD; *p* = 0.0017)	0.225 (η^2^)	4.900 to 12.40
IL-17A (pg/mL)	10.05 (4.08)	6.13 (1.44)	12.19 (1.433)	<0.0001 * (controls vs. SSVD; *p* < 0.0001 and AD vs. SSVD; *p* = 0.0018)	0.548 (η^2^)	−7.200 to −5.000 (controls vs. SSVD) and −5.600 to −1.300 (AD vs. SSVD)
IL-6/IL-17A	0.4251 (0.035)	1.179 (0.68)	3.181 (2.94)	<0.0001 * (controls vs SSVD; *p* < 0.001 and AD vs. SSVD; *p* = 0.0243)	0.422 (η^2^)	1.112 to 2.284 (controls vs. SSVD) and 0.3582 to 2.097 (AD vs. SSVD)
WBC	1.7 (2.7)	2.8 (2.4)	1.1 (2)	N/S	N/A	N/A

Abbreviations: SSVD: Subcortical small-vessel vascular dementia, AD: Alzheimer’s disease, WBC: white blood cells, CSF: cerebrospinal fluid, CRP: C-reactive protein, CHOL: cholesterol, LDL: low-density lipoprotein, TRIG: triglycerides, N/A: non-applicable. Data are represented as mean and SD (standard deviation). Comparisons among the two groups were performed with the Mann–Whitney U test. ∗ Kruskal–Wallis test and Dunn’s correction for multiple comparisons test are depicted among three group comparisons, and η^2^: eta squared.

## Data Availability

All data are reported within the article and are available (anonymized) by request from the qualified investigators.

## Supplementary Materials

The following supporting information can be downloaded at: https://www.mdpi.com/article/10.3390/diagnostics15060669/s1, Figure S1: CSF IL-6 and IL-17 levels in AD patients with various Fazekas scores.
