# Supplementary material for: Exploratory Analysis of Cerebrospinal Fluid IL-6 and IL-17A Levels in Subcortical Small-Vessel Disease Compared to Alzheimer’s Disease: A Pilot Study
Source: Diagnostics (Basel). 2025 Mar 10;15(6):669. doi: 10.3390/diagnostics15060669 (PMC11941723; doi:10.3390/diagnostics15060669)
Supplement: Supplementary file 1 [file diagnostics-15-00669-s001.zip › diagnostics-3404759-supplementary.pdf]

Supplementary Figure 1

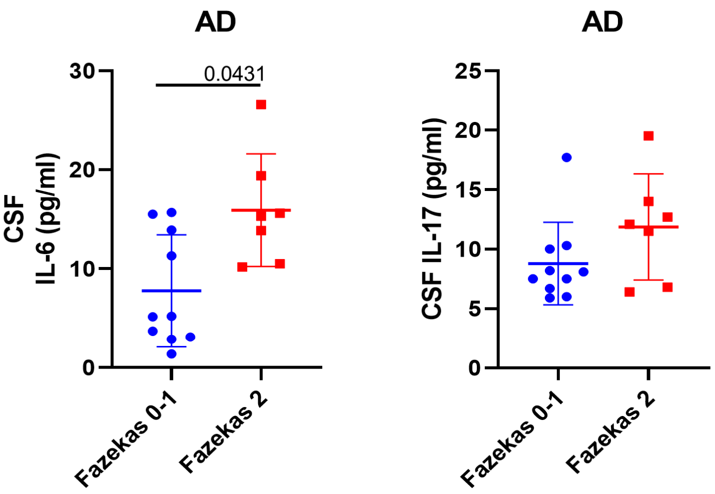

Supplementary Figure S1. CSF IL-6 and IL-17 levels in AD patients with various Fazekas scores. AD; Alzheimer's disease.
